# Gene Expression Modulation in Bovine Endometrial Cells Infected with Gammaherpesvirus Type 4 and Exposed to Lipopolysaccharide in the Presence of Platelet-Rich Plasma

**DOI:** 10.3390/v17060744

**Published:** 2025-05-23

**Authors:** Sofía López, Ignacio Álvarez, V. Andreoli, S. Delgado, S. Perez, S. Pereyra, F. Romeo, S. Grolli, Andrea Elizabeth Verna

**Affiliations:** 1 Instituto Nacional de Tecnología Agropecuaria, Instituto de Innovación para la Producción Agropecuaria y el Desarrollo Sostenible, Ruta 226, km 73.5, Balcarce B7620, Buenos Aires, Argentina; lopez.sofia@inta.gob.ar (S.L.);; 2 Division of Ruminant Medicine, Department of Clinical Sciences, Swedish University of Agricultural Sciences, P.O. Box 7054, 756 51 Uppsala, Sweden; ignacio.alvarez@slu.se; 3 Dipartimento di Scienze Agrarie, Forestali e Alimentari, Università di Torino, Largo Paolo Braccini, 2, 10095 Torino, Italy; 4 Facultad de Ciencias Agrarias, Universidad Nacional de Mar del Plata, Ruta 226, km 73.5, Balcarce B7620, Buenos Aires, Argentina; 5 Facultad de Ciencias Veterinarias, Universidad Nacional del Centro de la Provincia de Buenos Aires, Centro de Investigación Veterinaria de Tandil (CIVETAN), CONICET-UNCPBA-CICPBA, Campus Universitario, Tandil B7000GHG, Buenos Aires, Argentina; 6 Dipartimento di Scienze Medico Veterinarie, Università di Parma, Via del Taglio 8, 43126 Parma, Italy

**Keywords:** platelet-rich plasma, cattle, regenerative medicine, Bovine Gammaherpesvirus type 4

## Abstract

Uterine diseases in cattle are frequently linked to bacterial infections, with pathogens commonly isolated from the uterine lumen. Bovine Gammaherpesvirus Type 4 (BoGHV-4) is notably prevalent in certain regions of Argentina and is associated with uterine diseases in postpartum cattle. This study aims to evaluate the impact of platelet-rich plasma (PRP) on the gene expression related to BoGHV-4 infection in the presence of lipopolysaccharide (LPS), exploring the potential of PRP as a therapeutic alternative. The interaction between LPS and Toll-like receptor 4 (TLR4) plays a crucial role in inflammatory responses, triggering cytokine production and immune activation. Our results show that PRP modulates TLR4 and TNF-α gene expression, indicating a potential inhibitory role in inflammatory processes. Furthermore, PRP alter the temporal dynamics of BoGHV-4 replication by modulating the expression of the viral immediate–early gene (IE-2) and delaying proinflammatory cytokine responses such as IL-8. Notably, PRP enhances IFN-γ expression, which could help prevent tissue damage caused by bacterial and viral coinfection. These findings highlight the potential of PRP as an anti-inflammatory agent with therapeutic benefits in treating uterine diseases, offering an alternative to traditional antibiotic treatments.

## 1. Introduction

Argentine cattle farming faces the challenge of improving production efficiency to meet the growing global and local demand for food. Optimizing reproductive rates is crucial for increasing meat production per unit of land. Reproductive diseases, such as bovine endometritis, significantly impact reproductive efficiency by reducing conception rates, prolonging the interval between calving and conception, and increasing culling rates, all of which elevate production costs [1]. Endometritis is characterized by localized inflammation of the endometrial mucosa, edema, increased stromal cell density, and plasma cell infiltration into the stroma [2]. These alterations can negatively affect the endometrial microenvironment, thereby impairing the reproductive performance of the animals [3,4]. Although endometritis is a multifactorial disease, it can be triggered by pathogenic bacteria and viruses. Endometrial bacteria, such as *Escherichia coli* (*E. coli*), contain lipopolysaccharide (LPS) in their cell walls, a highly bioactive component that induces significant systemic inflammatory responses [5]. On the other hand, viruses such as Bovine Gammaherpesvirus type 4 (BoGHV-4) [6,7] reach the uterus through the bloodstream after infection and primarily affect the endometrial stroma. BoGHV-4 is a member of the *Gammaherpesvirinae* subfamily and the *Rhadinovirus* genus [8], distinguished by its ability to replicate in a wide range of species and cell cultures [9,10,11]. BoGHV-4 primarily infects B and T lymphocytes and exhibits a particular tropism for the endometrium, where it induces the death of epithelial and stromal cells [12,13]. Although its natural host is cattle and it is prevalent in the global livestock population, the pathogenic role of BoGHV-4 remains uncertain, as it has been identified in both asymptomatic animals and those exhibiting various clinical signs without being recognized as the etiological agent of a specific pathology [14,15,16,17,18,19]. BoGHV-4 was first detected in Argentina by Verna and colleagues in samples from cows with a history of abortion. These isolates exhibited high genetic variability, leading to the identification of 07-435 strain as genotype 3 [20]. This strain could be neutralized by sera from various ruminant species [21], highlighting its capacity to induce a neutralizing humoral response in the host, unlike other strains that show low efficiency in producing neutralizing antibodies [22,23].

The experiments conducted in vitro have demonstrated that bovine endometrial epithelial and stromal cells can respond to bacterial LPS through Toll-like receptors (TLRs), particularly TLR4 [24]. Activated TLRs stimulate macrophages to produce tumor necrosis factor-alpha (TNF-α) [25]. In animals with latent BoGHV-4 infection concomitant with bacterial LPS, the expression of IE-2 is stimulated, reactivating viral replication [12,26]. Donofrio et al. (2004) demonstrated that the expression of the IE-2 gene plays a crucial role in the initiation of lytic viral replication, not only during the reactivation of latency but also during de novo infections of permissive cells [9]. The expression of IE-2 leads to the production of ORF50/Rta, which transactivates the promoter of the interleukin-8 (IL-8) gene, a cytokine associated with neutrophil recruitment during inflammatory processes in epithelial and stromal endometrial cells. In immunocompetent animals, BoGHV-4 replication is controlled by the production of interferon-gamma (IFN-γ), which dysregulates IE-2, thereby inhibiting viral replication [27]. However, in animals with active BoGHV-4 replication and an altered IFN-γ response due to the presence of inflammatory molecules and pathogens, viral replication becomes uncontrolled and further stimulated [28].

Exploring various aspects of infection and immunity in the bovine genital tract will contribute to the development of new treatments and prevention strategies for uterine diseases [29]. Currently available treatments primarily rely on antibiotics to counteract infection and subsequent excessive inflammation at the site [30], without addressing the endometrial regeneration process. However, these treatments may have adverse effects on cow fertility [31,32]. Therefore, alternative therapies based on the use of biomolecules, such as platelet-rich plasma (PRP), are crucial from a “One Health” perspective. PRP is obtained by centrifuging whole peripheral blood collected in the presence of anticoagulants [33]. This autologous blood product is characterized by high concentrations of platelets, active metabolites, and growth factors [34,35,36], which work synergistically to activate anti-inflammatory and regenerative pathways, thereby resolving persistent pathological states [37].

Therefore, this study aimed to evaluate the modulatory effect of PRP on the expression of genes associated with BoGHV-4 infection in endometrial cells in the presence of LPS, considering its potential application as a therapeutic alternative due to its high concentration of growth factors.

## 2. Materials and Methods

### 2.1. Culture of Bovine Endometrial Cells (BECs)

The primary culture of endometrial cells was obtained from bovine uteri without signs of genital disease, following the protocol described by Romeo et al., 2021 [38]. The cells were cultured in minimum essential medium with Earle’s salts (MEM-E, Gibco; Thermo Fisher Scientific, Carlsbad, CA, USA) supplemented with antibiotics and antimycotics [38] in the presence of commercial fetal bovine serum (FBS; Bioser, Buenos Aires, Argentina) in a humidified atmosphere at 37 °C until confluence was reached. To exclude the presence of other pathogens, such as Bovine Alphaherpesvirus 1 (BoAHV-1), Bovine Bammaherpesvirus type 4 (BoGHV-4), and Bovine Viral Diarrhea virus (BVDV), antigen detection was performed via direct immunofluorescence (DIF) with a porcine polyclonal antiserum (VMRD, Inc., Pullman, WA, USA). Furthermore, nucleic acid detection via nested PCR or RT‒PCR and viral isolation were performed [38,39,40].

### 2.2. Viral Infection

The 07-435 strain of BoGHV-4, isolated from the cervical–vaginal mucus of a cow with a history of abortion in Argentina, was used in this study. The viral strain was propagated in the Madin–Darby Bovine Kidney (MDBK) cell line cultured in T-25 flasks (Greiner Bio-One, Numbrecht, Germany) at a density of 1 × 10^5^ cells/mL for 48 h. Viral titration was performed using the endpoint dilution method with MDBK cells grown in 96-well microtiter plates (Greiner Bio-One, Numbrecht, Germany). The viral titers were determined at 72 h post-infection (hpi) and expressed as the 50% tissue culture infectious dose per milliliter (TCID_50_/mL) [41].

### 2.3. Preparation of Platelet-Rich Plasma (PRP)

PRP was obtained from the peripheral blood of donor bovine provided by the INTA Institution, following the guidelines outlined in the Institutional Committee for the Care and Use of Experimental Animals (CICUAE) protocol (232/2021). Blood collection was performed using 4% sodium citrate as an anticoagulant. PRP was prepared using a semi-automated closed system (Ematik^®^ SemiManual Kit, Prometheus Srl, Parma, PR, Italy), strictly adhering to the manufacturer’s instructions. After two centrifugation cycles, platelets were resuspended in a small volume of platelet-poor plasma (PPP), which was obtained as the supernatant after the second centrifugation. The final PRP concentration was adjusted to achieve a platelet count between 800 × 10^6^ and 1.3 × 10^9^ platelets/mL. The prepared PRP was stored at −20 °C until further use [42]. The blood from the donor animal was tested to rule out the presence of pathogens: BVDV, BoHV-1, BoHV-5, BoGHV-4, Brucella, and *E. coli*. The results were negative according to the technique applied.

### 2.4. Viral Infection and PRP Treatment

**Figure 1:**
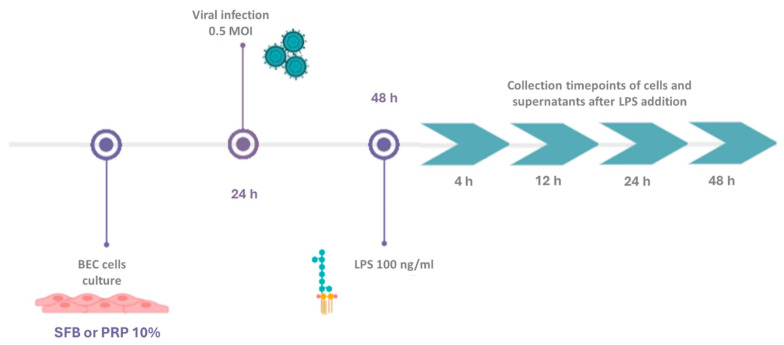
Experimental design of BECs grown in media supplemented with FBS or PRP and infected with the BoGHV-4 strain 07/435 in the presence or absence of LPS.

To evaluate the effect of PRP on the expression of genes related to BoGHV-4 infection genes in the presence of LPS a total of 800,000 BECs were cultured in a 6-well plate (Greiner Bio-One, Numbrecht, Germany) in the presence of fetal bovine serum (FBS) or 10% PRP supplemented with antibiotics (penicillin 100 IU/mL, streptomycin 100 µg/mL) and antifungals (25 µg/mL of amphotericin B) (Gibco, Grand Island, NY, USA) at 37 °C in a 5% CO_2_ atmosphere. After 24 h, when the cells reached confluence, they were infected by adsorption at 0.5 MOI with the 07-435 strain of BoGHV-4. Considering the relevance of LPS in postpartum uterine infections [29,43,44] and its presence in clinical cases of bacterial coinfections with BoGHV-4, 24 h after viral infection, a concentration of 100 ng/mL LPS (LPS *E. coli* O55. B5; Santa Cruz Biotechnology, Inc., Dallas, TX, USA) was added to the medium as described by Chanrot et al., 2017 and Shen et al., 2018 [45,46]. LPS was maintained in the culture medium throughout the experiment. Cells and supernatants were collected at 4, 12, 24, and 48 h. Each plate included a negative control with uninfected BECs, cells infected with the virus alone, and cells treated with LPS alone. Each sample was analyzed in triplicate (Figure 1).

### 2.5. RT‒qPCR

To evaluate the expression of genes involved in BoGHV-4 replication and inflammatory cellular processes, the relative expression levels of the TLR4, TNF-α, IL-8, IFN-γ, IE-2, and bovine glyceraldehyde-3-phosphate dehydrogenase (GAPDH) genes were determined in BEC cultures by RT‒qPCR.

For this purpose, the collected BECs were stored in BIO-ZOL Reagent (PB-L, Argentina) at −80 °C for subsequent RNA extraction, following the manufacturer’s instructions. On average, 0.5 μg of total RNA was used for first-strand cDNA synthesis (iScript™, Bio-Rad Laboratories, Inc., Hercules, CA, USA) according to the provider’s protocol. The cDNA was stored at −80 °C until RT‒qPCR was performed with SsoAdvanced Universal SYBR Green Supermix (Bio-Rad Laboratories), following the manufacturer’s instructions. Samples were analyzed using the CFX96 Touch thermocycler (Bio-Rad Laboratories). Amplification was performed under the following conditions: 10 min at 95 °C, 40 cycles of 15 s at 95 °C and 1 min at 60 °C. The primers used for the assay are detailed in Table 1. All the samples were amplified in triplicate, and the RT‒qPCR products were expressed as threshold cycle (Ct) values. The expression of the gene of interest was normalized to that of the endogenous gene GAPDH.

### 2.6. Statistical Analysis

The data were collected under a completely randomized design with two replications for each combination between, on one hand, TLR4, TNF-α, IL-8 and IFN-γ and, on the other hand, the FBS and PRP cultures. For each combination mentioned above, the expressions of RT‒qPCR were analyzed by fitting a statistical model with two factors. There were twelve treatments in total, and they arose from the combination of virus, LPS and LPS + virus levels, with the 4 different times (4, 12, 24, and 48 h). Using an analysis of variance, the statistical hypothesis that states the absence of interaction between time levels and treatments with virus, LPS and LPS + virus was tested. Comparisons of means between virus treatments for each time level were then performed using Fisher’s protected test with the significance level adjusted by the Bonferroni method.

Under the same experimental design, for IE-2 the RT‒qPCR expressions were collected for each combination between the FBS and PRP cultures and time levels (4, 12, and 24 h). In this case, there were six treatments, consisting of a time level and a culture level. The statistical hypothesis that states the absence of interaction between time levels and the culture was tested. Comparisons of means between the cultures for each time level were then performed using Fisher’s protected test with the significance level adjusted by the Bonferroni method.

The computational support used to perform the statistical analysis was R program version 4.4.0 (R Core Team, 2024). In all hypothesis tests, the significance level used was five percent (α = 0.05).

## 3. Results

**Figure 2:**
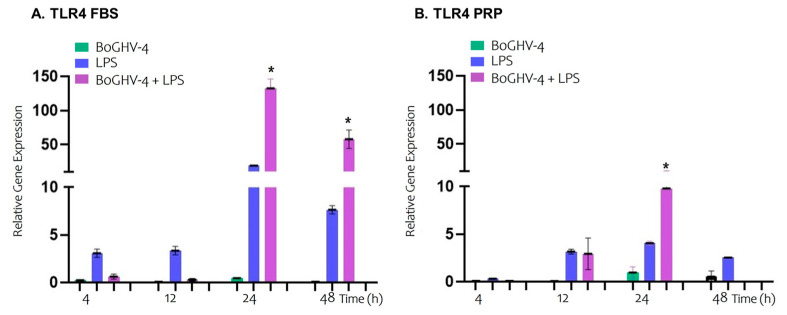
Relative expression of the TLR4 gene normalized to that of GAPDH at different time points. Panels (**A**,**B**) represent BECs grown in FBS and PRP, respectively. The symbols * represent statistically significant differences (*p* < 0.05).

In Figure 2A,B, the relative expression of the TLR4 gene in BECs cultured in the presence of FBS or PRP at different time points is shown.

Significant changes in mRNA expression of TLR4 in BECs infected with BoGHV-4 were not detected over the time points evaluated. However, when the cells were incubated with LPS, a slight, although non-significant increase in TLR4 expression was observed over time. In contrast, in the cells infected with the virus and incubated with LPS, a significant increase (*p* < 0.05) in the relative expression of the gene was observed, with 132.5- and 57.7-fold increase at 24 and 48 h, respectively.

Similarly to what was observed in BECs cultured with FBS, no significant differences were found in BECs treated with PRP, either infected with BoGHV-4 or incubated with LPS, at the analyzed time points. However, in the cells infected and incubated with LPS, a significant increase (9.78-fold) (*p* < 0.05) in TLR4 expression was observed at 24 h, followed by inhibition of gene expression at 48 h.

**Figure 3:**
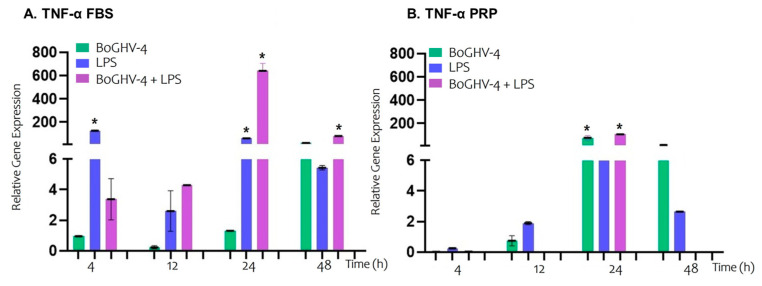
Relative expression of the TNF-α gene normalized to that of GAPDH at different time points. Panels (**A**,**B**) represent BECs grown in FBS and PRP, respectively. The symbols * represent statistically significant differences (*p* < 0.05).

When the relative expression of the TNF-α gene (Figure 3A) in BECs infected with BoGHV-4 cultured in FBS-supplemented medium was analyzed, no significant increases were detected at the evaluated time points. However, when the cells were incubated with LPS, a significant increase (*p* < 0.05) in gene expression was recorded, reaching values 126.00 and 60.85 times greater at 4 and 12 h, respectively. In the case of coinfection with the virus and LPS, a significant increase (*p* < 0.05) was observed at later time points, with expression levels 641.76 and 79.96 folds higher at 24 and 48 h, respectively.

On the other hand, when BECs infected with BoGHV-4 were cultured in medium enriched with PRP (Figure 3B), a significant increase (*p* < 0.05) in gene expression of 75.63 times was observed at 24 h, with no significant changes at the other evaluated time points. The genes expressed in the cells exposed to LPS did not significantly differ at any of the time points analyzed. Similarly to what was observed in infected cells, those coinfected with the virus and incubated with LPS presented a significant increase (*p* < 0.05) in TNF-α expression at 24 h, (10.18-fold higher). However, at 48 h, gene expression was inhibited.

**Figure 4:**
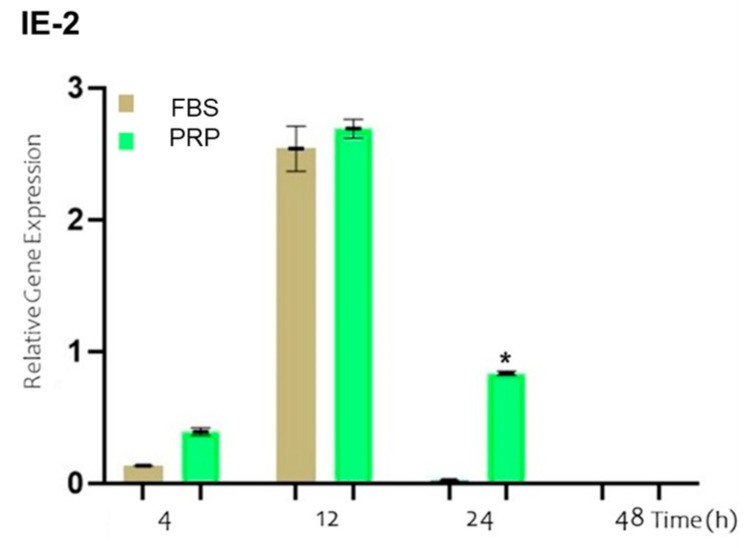
Relative expression of the viral IE-2 gene normalized to that of GAPDH in BECs co-incubated with BoGHV-4 and LPS at different time points. The symbols * represent statistically significant differences (*p* < 0.05).

The expression of the IE-2 gene is one of the first events that occurs following the entry of the virus into the host cell. In Figure 4, the results of the relative expression levels of IE-2 at the studied time points are shown for cells incubated with BoGHV-4 and LPS and cultured in either FBS-supplemented medium (brown bar) or PRP-supplemented medium (green bar). No significant changes in the expression of the IE-2 gene were detected at 4 and 12 h. However, at 24 h, a significant increase (*p* < 0.05) of 0.8 times in gene expression was evident when the cells were cultured in PRP-supplemented medium compared with those cultured in FBS-supplemented medium.

**Figure 5:**
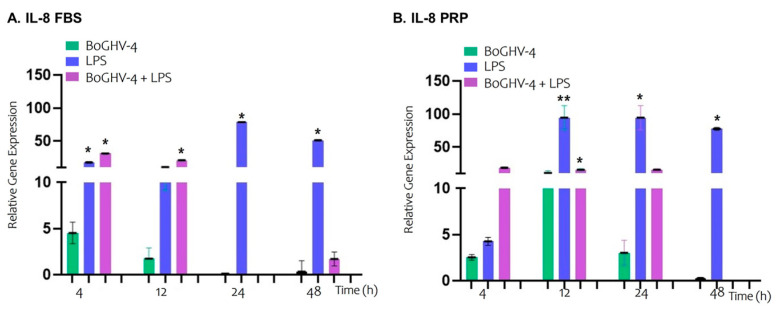
Relative expression of the IL-8 gene normalized to that of GAPDH at different time points. Panels (**A**,**B**) represent BECs grown in FBS and PRP, respectively. The symbols * represent statistically significant differences (*p* < 0.05) and symbols ** represent statistically significant differences (*p* < 0.05) between the average from * and the one without *.

In the analysis of the relative expression of the IL-8 gene in BECs infected with BoGHV-4 cultured in FBS-supplemented medium (Figure 5A), no significant changes were observed at the different evaluated time points. However, when the cells were incubated with LPS, a significant increase (*p* < 0.05) in IL-8 expression was recorded at 4 h (17.52-fold), 24 h (78.43-fold), and 48 h (51.05-fold). On the other hand, when the cells were co-incubated with BoGHV-4 and LPS, IL-8 expression significantly increased (*p* < 0.05) at earlier time points, specifically at 4 h (30.96-fold) and 12 h (20.78-fold).

In the case of BECs infected with BoGHV-4 cultured in PRP-supplemented medium (Figure 5B), no significant changes in IL-8 gene expression were observed at the time points studied. However, in the cells incubated with LPS, significant increases (*p* < 0.05) of 94.31, 94.52, and 77.48 times were observed at 12, 24, and 48 h, respectively. In contrast, co-incubation of the cells with BoGHV-4 and LPS in PRP-supplemented medium resulted in a significant increase (*p* < 0.05) only at 12 h, (15.52-fold higher).

**Figure 6:**
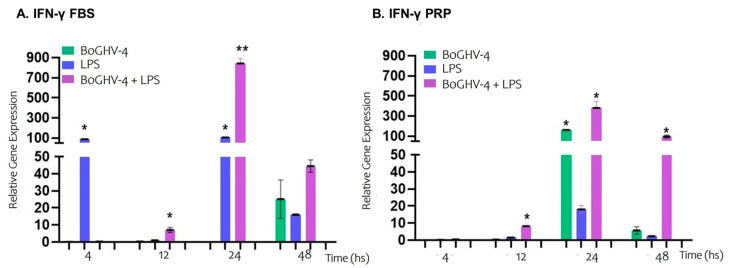
Relative expression of the IFN-γ gene normalized to that of GAPDH at different time points. Panels (**A**,**B**) represent BECs grown in FBS and PRP, respectively. The symbols * represent statistically significant differences (*p* < 0.05) and symbols ** represent statistically significant differences (*p* < 0.05) between the average from * and the one without *.

In Figure 6A, the relative expression of the IFN-γ gene in BECs grown in FBS-supplemented medium are presented. Cells infected with BoGHV-4 did not significantly modify the expression of IFN-γ at the evaluated time points. However, in cells incubated with LPS, gene expression increased significantly (*p* < 0.05) by 86.98- and 103.93-fold at 4 and 24 h, respectively. In contrast, when the cells were co-incubated with the virus and LPS, gene expression significantly increased by 7.04- and 842.15-fold at 12 and 24 h, respectively.

On the contrary, when the cells were grown in PRP-supplemented medium (Graph 6B), the gene expression of the cells infected with BoGHV-4 significantly increased (*p* < 0.05) by 161.04 times at 24 h but not at the other evaluated time points. However, in cells incubated with LPS, the expression of IFN-γ was not modified. In cells co-incubated with the virus and LPS, there was a significant increase (*p* < 0.05) of 8.20, 379.85, and 94.81 times at 12, 24, and 48 h, respectively.

## 4. Discussion

Most uterine diseases are associated with bacterial infections, as several of these pathogens are commonly isolated from the uterine lumen [7,29,47,48]. Additionally, BoGHV-4, which has a high prevalence in certain regions of Argentina [49], is consistently associated with uterine diseases in postpartum cattle. In BoGHV-4 persistently infected cattle, the virus remains latent in macrophages. A theoretical model has been proposed based on bacterial–virus coinfection, where persistently infected macrophages are recruited from the bloodstream to sites of inflammation in the endometrium, leading to the activation of viral replication [13,50]. Tebaldi et al. (2016) [51] proposed a model from a deep transcriptomic analysis that explains the molecular mechanisms occurring in the endometrium in response to bacterial infection in animals chronically infected with BoGHV-4. When pathogens involved in the onset of uterine disease are detected, they are generally recognized by Toll-like receptors (TLRs) and are rapidly eliminated through the activation of signalling cascades and subsequent pathways. The stimulation of this initial defence mechanism in the endometrium leads to the synthesis and production of a wide range of proinflammatory cytokines and chemokines, which, in turn, mobilize and activate immune system cells [29].

In cattle, different therapeutic protocols have been developed for uterine diseases, including systemic or intrauterine infusion of antibiotics [52,53]. However, these treatments are associated with potential adverse effects on future fertility [31,32]. For these reasons, developing new therapeutic approaches to treat uterine diseases safely and effectively while also considering alternatives based on the “One Health” concept is crucial. In recent years, biotherapy with platelet-rich plasma (PRP) has gained increasing scientific attention and support in the treatment of uterine diseases because of its natural anti-inflammatory and antimicrobial properties. This autologous biological product represents a natural mixture of active metabolites and growth factors that synergistically contribute to the activation of regenerative physiological pathways to resolve persistent pathological states [37]. In light of these findings, the aim of the present study was to evaluate the effect of PRP on the expression of genes related to BoGHV-4 infection in the presence of LPS, considering its potential use as a therapeutic alternative owing to its high abundance of growth factors.

The interaction between TLR4 and LPS plays a crucial role in the inflammatory response. When LPS binds to TLR4, it activates a signalling cascade that ultimately leads to the activation of viral IE-2 and triggers the expression of various proinflammatory cytokines, such as TNF-α and IL-8 [54,55]. This signalling pathway in normogenic animals may be controlled by IFN-γ [28]. In summary, this axis represents a key mechanism through which inflammation induced by LPS and BoGHV-4 exacerbates tissue dysfunction and promotes the cycle of inflammation and immune activation [56].

The results of the relative expression of the TLR4 gene in BECs infected with BoGHV-4 or incubated with LPS revealed no significant changes at the evaluated time points when the cells were grown in either FBS or PRP medium. However, the gene was stimulated at 24 and 48 h in BECs co-incubated with the virus and LPS in FBS medium, whereas with PRP, it was stimulated at 24 h followed by inhibition at 48 h. These results could indicate that PRP negatively modulates the expression of TLR4 at prolonged time points in cells exposed to BoGHV-4 and LPS.

Herath and colleagues proposed that the activation of TLRs triggers the stimulation of macrophages to produce TNF-α [57]. In line with these findings, our results show that the relative expression of the TNF-α gene in BEC infected with BoGHV-4 and grown in FBS did not significantly change. In contrast, PRP-supplemented medium promoted TNF- α gene expression, whereas incubation with LPS in FBS-supplemented medium significantly increased gene expression. However, this response was not observed in cells cultured with PRP, suggesting a potential modulatory role of this component in the regulation of proinflammatory molecules such as TNF-α. Similarly to what was observed for TLR4, in BECs co-incubated with the virus and LPS and grown in FBS medium, TNF-α gene expression significantly increased at 24 and 48 h, whereas in PRP medium, gene expression was suppressed at 48 h. These results highlight the potential negative effects of PRP on the inflammatory response mediated by the expression of cytokines such as TNF-α.

The efficient replication of BoGHV-4 in endometrial cells has been associated with post-entry events, including the transactivation of the IE-2 gene promoter [12]. Proinflammatory molecules such as TNF-α and LPS have been reported to enhance gene expression, thereby promoting viral replication and dissemination within the uterine stroma [13,58]. In this study, we observed that the microenvironment generated by PRP not only modulated IE-2 gene expression but also altered its temporal dynamics compared with FBS. These findings suggest that PRP may play a pivotal role in regulating viral replication, potentially through the modulation of the inflammatory response, which could have implications for BoGHV-4 pathogenesis and the development of alternative therapeutic strategies.

The experiments conducted in vitro demonstrated that TNF-α produced by LPS-stimulated macrophages induces the expression of the IE-2 of BoGHV-4, whose product (ORF50/Rta) transactivates the promoter of the IL-8 gene, a chemokine associated with neutrophil attraction in inflammatory processes during the infection of epithelial and endometrial stromal cells [59]. Gene expression assays of IL-8 in BECs infected with BoGHV-4 revealed no significant changes over time when the cells were grown in either FBS or PRP media. However, when BECs were exposed to LPS, IL-8 gene expression was significantly increased at early time points (4 h) in FBS medium, whereas with PRP, this effect was observed after 12 h. This same pattern was observed when the cells were co-incubated with both the virus and LPS. These results highlight that PRP delays the expression of proinflammatory molecules to later time points [59].

Tebaldi and colleagues demonstrated that IFN-γ can inhibit BoGHV-4 replication in BECs through the negative regulation of the IE-2 promoter [51]. Consistent with the well-established role of IFN-γ in suppressing viral reactivation, our findings revealed that in BECs infected with BoGHV-4 and cultured in FBS-supplemented medium, IFN-γ expression remained stable. However, a significant increase in IFN-γ expression was observed when cells were cultured in PRP-supplemented medium at 24 h post-treatment. In contrast, exposure to LPS in FBS-supplemented medium resulted in a marked upregulation of IFN-γ gene expression at both 4 and 24 h, whereas no significant changes were detected in PRP-supplemented medium under the same conditions. Furthermore, when BECs were co-incubated with both the virus and LPS in FBS medium, there was a significant increase in gene expression at 12 and 24 h, whereas in PRP medium, this effect was prolonged up to 48 h. These results could indicate that PRP favours prolonged expression of IFN-γ, thereby preventing inflammation and tissue damage resulting from combined viral and bacterial infection.

These results demonstrate that PRP has a significant effect on the signalling pathways associated with coinfection by *E. coli* and BoGHV-4, as described by Jacca et al., 2014 [28], highlighting the potential of PRP as an anti-inflammatory agent in the treatment of uterine diseases. The positive effect observed in this study could be attributed to the richness of bioactive factors, such as cytokines and growth factors, which have potential for modulating cellular inflammation—key processes for controlling viral replication and the host response. However, further research is essential to elucidate the underlying molecular and protein mechanisms, as well as to optimize their application in the development of biomolecule-based treatments, which could provide an effective alternative to antibiotic use.

## Figures and Tables

**Table 1:** Primers used for gene expression evaluation via RT‒qPCR.

Primer Sequences		
Name	Type	Sequences
GAPDH	FWD	5′-TTC TGG CAA AGT GGA CAT CGT-3′
GAPDH	REV	5′-CCT GAC TGT GCC GTT GAA CTT-3′
IL-8	FWD	5′-CCT CTT GTT CAA TAT GAC TTC CA-3′
IL-8	REV	5′-GGC CCA CTC TCA ATA ACT CTC-3′
TNF-α	FWD	5′-CCA CGT TGT AGC CGA CAT CA-3′
TNF-α	REV	5′-CTG GTT GTC TTC CAG CTT CAC A-3′
IE-2	FWD	5′-ACA AAC ACA CAG ACC AGT CA-3′
IE-2	REV	5′-GTT TCA CAA CAG ATT GAG CA-3′
TLR4	FWD	5′-TGC CTT CAC TAC AGG GAC TTT-3′
TLR4	REV	5′-AGT GTC GCT GTT GAA GTC-3′
IFN-γ	FWD	5′-CAG CTC TGA GAA ACT GGA GGA CTT-3′
IFN-γ	REV	5′-GTG GCT GGA GTG GTT ATT AG-3′

## Data Availability

Data are contained within the article.

## Abbreviations

The following abbreviations are used in this manuscript:

BoGHV-4	Bovine Gammaherpesvirus Type 4
PRP	Platelet-rich plasma
LPS	Lipopolysaccharide
TLR4	Toll-like receptor 4
IE-2	Viral immediate–early gene
TNF-α	Tumor necrosis factor-alpha
IL-8	Interleukin-8
IFN-γ	Interferon-gamma
BEC	Bovine endometrial cells
FBS	Fetal bovine serum
BoAHV-1	Bovine Alphaherpesvirus 1
BVDV	Bovine Viral Diarrhea virus
MDBK	Madin–Darby Bovine Kidney
hpi	hours post-infection
PPP	Platelet-poor plasma
GAPDH	Glyceraldehyde-3-phosphate dehydrogenase
Ct	threshold cycle
